# Improving Diuretic Response in Heart Failure by Implementing a Patient-Tailored Variability and Chronotherapy-Guided Algorithm

**DOI:** 10.3389/fcvm.2021.695547

**Published:** 2021-08-11

**Authors:** Ariel Kenig, Yotam Kolben, Rabea Asleh, Offer Amir, Yaron Ilan

**Affiliations:** ^1^Department of Medicine, Hebrew University-Hadassah Medical Center, Jerusalem, Israel; ^2^Department of Cardiology, Hebrew University-Hadassah Medical Center, Jerusalem, Israel; ^3^The Azrieli Faculty of Medicine in the Galilee, Bar-Ilan University, Safed, Israel

**Keywords:** heart failure, diuretic resistance, chronobiology, variability, digital systems

## Abstract

Heart failure is a major public health problem, which is associated with significant mortality, morbidity, and healthcare expenditures. A substantial amount of the morbidity is attributed to volume overload, for which loop diuretics are a mandatory treatment. However, the variability in response to diuretics and development of diuretic resistance adversely affect the clinical outcomes. Morevoer, there exists a marked intra- and inter-patient variability in response to diuretics that affects the clinical course and related adverse outcomes. In the present article, we review the mechanisms underlying the development of diuretic resistance. The role of the autonomic nervous system and chronobiology in the pathogenesis of congestive heart failure and response to therapy are also discussed. Establishing a novel model for overcoming diuretic resistance is presented based on a patient-tailored variability and chronotherapy-guided machine learning algorithm that comprises clinical, laboratory, and sensor-derived inputs, including inputs from pulmonary artery measurements. Inter- and intra-patient signatures of variabilities, alterations of biological clock, and autonomic nervous system responses are embedded into the algorithm; thus, it may enable a tailored dose regimen in a continuous manner that accommodates the highly dynamic complex system.

## Introduction

Heart failure (HF) is a staggering clinical and public health problem with high morbidity and mortality burden, affecting more than 6 million individuals in the United States where ~670,000 individuals are diagnosed with HF each year (1). Patients with HF are frequently hospitalized with HF exacerbation and have reduced quality of life and increased mortality rates. HF exacerbation is the leading cause of hospitalization among patients aged >65 years (2).

HF is associated with high rates of morbidity and reduced quality of life which are related to symptoms of volume overload resulting from sodium retention and volume overload. Loop diuretics are commopnly used for the teratment of volume overload resulting from HF. There is a marked inter and intra-patient variability that characterizes the response to diuretics at different stages. These variations may alter patients' clinical course and adversely affect their clinical outcomes (3, 4). A diminished response to loop diuretics is a well-recognized clinical challenge, which limits their clinical use (5, 6). Current methods for dose adjustment are somewhat arbitrary and do not take into consideration patients' resistance to diuretics (6). A continuous increase in the diuretic doses may further worsen diuretic resistance.

In the present article, we review the common mechanisms underlying the development of diuretic resistance and discuss current methods to overcome this devastating phenomenon. The role of the autonomic nervous system (ANS) and chronobiology in the pathogenesis of congestive heart failure (CHF) are discussed. The implementation of a patient-tailored variability and chronotherapy-guided algorithm for the treatment of these patients is presented as a potential strategy for alleviating drug resistance and improving their long-term beneficial effects.

## Diuretic Resistance Is a Major Difficulty in the Treatment of HF

The effects of loop diuretics on systemic and renal hemodynamics are driven by multiple factors, including the dose, route of administration, concomitant diseases, and medications and chronicity of their use (7). Furosemide is a potent prototypic loop diuretic that exerts its effect by binding to the translocation pocket at the extracellular surface of sodium-potassium-chloride symporters (NKCCs). It blocks ion transport directly by inhibiting NKCCs at the apical surface of the thick ascending loop of Henle (7, 8). The half-life of loop diuretics is generally shorter than typical dosing intervals of twice daily. Additionally, as they inhibit solute transport at only a single segment out of the numerous sodium-reabsorbing nephron sections, their impacts on extracellular fluid volume are multifaceted. Loop diuretics cause renal vasodilation through direct vascular dilation of the afferent arterioles and inhibition of the tubuloglomerular feedback (9).

### Definition of Diuretic Resistance

Diuretic resistance is defined as the failure to achieve effective decongestion with low urine sodium concentration despite the use of appropriate or escalating diuretic doses (10). When diuretic resistance develops, the response to diretics is reduced before the achieving the goal of treatment, leading to poor prognosis. Resistance (or tolerance) to diuretic therapy can develop over time, making volume reduction in HF more challenging. There are two forms of diuretic tolerance, namely, short- and long-term resistance. Short-term tolerance occurs when the effect of the diuretic is weakened after the first dose. It may be prevented by reestablishing diuretic-induced loss of volume. Long-term tolerance is observed following administration of a loop diuretic for prolonged periods of time, which is associated with sodium reabsorption at the distal sites (10).

### Factor That Are Associated With Diuretic Resistance

Multiple factors can explain non-responsiveness to diuretics, including inadequate doses, lack of adherence, advanced age, high sodium intake, impaired secretion into the tubule lumen, chronic kidney disease (CKD), gut edema, use of non-steroidal anti-inflammatory agents, hypoproteinemia, hypotension, nephrotic syndrome, reduced renal blood flow, and neurohormonal activation (7, 11).

Tolerance at the receptor- or post-receptor points may be associated with diuretic tolerance or resistance (11). In the circulation, loop diuretics are bound to various proteins (mainly albumin) and secreted into the tubules by the organic anion transporters (OAT1 and OAT2) located at the basolateral membrane and multidrug resistant protein-4 located at the apical membrane. As diuretics compete with chloride for binding to NKCC2, increased salt absorption in the proximal tubules limits the diuretic-sensitive transport (7).

In patients with HF, RAAS activation results in sodium retention. These patients may manifet with renal dysfunction assciated with additional activation of neurohormones. As HF progresses, persistent activation of these neurohormonal systems enhances sodium retention and contributes to the development of diuretic resistance (12–14). Worsening renal function in HF reduces the usefulness of loop diuretics by reducing their secretion into the renal tubules, a process mediated by increased organic ions competing for organic ion transporter binding (15).

### Compensatory Responses to Diuretics

Compensatory responses to diuretics may increase drug resistance. The diuretic-associated increase of the plasma renin activity promotes angiotensin II while blocking the tubuloglomerular feedback for increasing the glomerular filtration rate (16). Angiotensin II stimulates proximal tubular sodium reabsorption, thereby reducing distal sodium delivery and leading to diuretic resistance (11). Elevated plasma levels of both angiotensin II and aldosterone activate sodium transporters in the distal nephron (17). Post-diuretic sodium retaining may occur once the loop diuretic concentration drops below a certain threshold in the renal tubules. Sodium reabsorption is increased in the distal tubules and collecting ducts. This effect counters the loop diuretic effects (14). Adding of a second or third daily dose of loop diuretic may overcome this effect but may be associated with further long-term tolerance.

### Distal Nephron Remodeling

An additional contributing factor to diuretic resistance is the “braking phenomenon.” Chronic use of high dosages of loop diuretics leads to distal nephron remodeling, which involves hypertrophy and hyperplasia of the distal convoluted and connecting tubules and the collecting duct. This process augments the reabsorption capacity of the distal nephron and results in enhanced sodium reabsorption that negates the anticipated beneficial effects of these diuretics (18–20). This process is partially mediated by the RAAS (21) and activation of the sympathetic nervous system (SNS) (22). Adding a thiazide diuretic that blocks sodium reabsorption in the distal tubules This occurrence can overcome this effect; thus, it augments the net loss of sodium. Activation of baroreceptors in the arterial system triggers the SNS and RAAS, nephron remodeling, and extracellular volume depletion, thereby resulting in sodium retention (23). Extracellular volume contraction, which occurs with prolonged diuretic use in the setting of persistent congestion, contributes to the development of drug resistance (7).

## Current Measures for Overcoming Diuretic Resistance

Several pharmacologic and non-pharmacologic interventions are being used in an attempt to improve the diuretic response, including intravenous administration, increasing diuretic doses, and changing diuretic agents (7, 11). Several of these are directly or indirectly related to continuous daily administration of similar or higher dosages of diuretics. However, despite the prevalence of diuretic resistance, there remains a paucity of clinical trials to provide evidence on how to mitigate the resistance and guide therapy when patients develop this phenomenon (24).

Increasing dosages of loop diuretics may lead to a plateau in their effect, suggesting that raising doses beyond a “ceiling” will not further augment response (7). High diuretic doses, which stimulate the RAAS and SNS, are associated with worse outcomes, raising the possibility that they should be avoided in patients with severe decompensated HF (25). However, the Diuretic Optimization Strategies Evaluation study demonstrated that patients with acute decompensated HF on higher diuretic doses showed more favorable outcomes, including significant relief of dyspnea, reduction in weight, and higher net fluid loss, although they had a higher incidence of worsening renal function. Notably, the initial elevation in serum creatinine levels was associated with improved clinical outcomes (26).

Combined diuretic therapy is increasingly used to overcome high sodium retention. A common way to overcome diuretic resistance is to combine a diuretic with other medications including mineralocorticoid receptor antagonists, acetazolamide, or metolazone (6). The sequential nephron blockade by combining loop and thiazide diuretics in patients with inadequate response to optimal doses of loop diuretics was reportedly effective in some studies (27). Dopamine can improve renal perfusion and exert a diuretic effect (28). Hypertonic saline transiently increases the intravascular volume and improves sodium delivery to the tubules of the nephron (28). Additionally, metalazone (zaroxylin) in combination with loop diuretics or ultrafiltration is also being used in patients with resistance (29, 30).

The CardioMEMS HF System was designed for monitoring and measuring pulmonary artery (PA) pressure in patients with CHF. It transmits daily sensor readings from patients to their healthcare providers, allowing for personalized diuretic management in order to reduce the likelihood of hospitalization. In a retrospective analysis, PA pressure-guided management reduced HF hospitalization by 43% and mortality by 57% (31–33). The CHAMPION trial was a controlled, single-blind study of 550 patients with New York Heart Association class III HF and an HF hospitalization in the previous year. After 6 months of follow-up, the PA sensor-actively monitored patients who experienced an increased frequency of medication adjustments, higher dosages of diuretics, and diuretics intensification (34). Compared with the standard of care, its use was cost-effective, with an incremental cost-effectiveness ratio per quality-adjusted life year (31). However, dose adjusting is marginally arbitrary and does not consider patients developing resistance to diuretics.

Taken together, similar mechanisms are responsible for both the resistance and adaptation to diuretics. Several measures taken for overcoming diuretic resistance may further enhance the vicious cycle of inducing resistance and jeopardizing the clinical condition. Continuous administration on a regular basis of the same or higher dosages of diuretics is associated with a vicious cycle of actually augmenting the resistance.

## Disruption of the Circadian Rhythm in HF

Chronobiology describes the control of multiple biological functions by the circadian rhythm. Many cellular and physiological processes exhibit a circadian rhythm, oscillating approximately once in 24 h. These endogenous cycles enable the organism to optimally arrange its patterns of behavior in synchronization with the predictable changes in environmental conditions (35, 36). The central pacemaker is loated in the suprachiasmatic nuclei (SCN) and is synchronized with multiple peripheral clocks in various cells (37). In the cellular level, four genes—*circadian locomotor output cycle kaput* (*clock), brain and muscle ARNT-like 1 (bmal1), period 1 and 2 (Per1,2)*, and *Cryptochrome (Cry)—*form a transcriptions-translation feedback loop that cycles approximately every 24 h, providing the periodicity of the cellular circadian rhythm.

### Daily Oscillations in Heart and Blood Vessels

The heart and blood vessels are characterized by marked daily oscillations in gene expression, metabolism, growth, and remodeling. The circadian clocks within the cardiomyocytes are linked to the regulation of myocardial function and metabolism (38). A proper response of the heart to its diurnal environment is mandatory for survival. It involves response to changes in workload, nutrients, neurohumoral stimuli, and metabolic alterations. Blood pressure (BP), heart rate (HR), coagulation activity, and endothelial function manifest in a day/night pattern (39).

### Chrono Disruption of the Circadian Rhythm

Chronodisruption is an alteration of circadian rhythms associated with diverse diseases, including cardiovascular diseases and malignancy. Night-shift workers, who are exposed to artificial light disrupting the endogenous circadian rhythm, have a higher risk of all-cause and cardiovascular mortality (40). Obesity, high triglycerides, and low high-density lipoprotein cholesterol levels are more common in night-shift workers (41). Brachial artery endothelial function is determined as the reaction to reactive hyperemia (flow-mediated dilation [FMD]) and was irregular in residents following a 24-h shift, including night duty. A marked decrease in FMD was noted in physicians with a history of night-shift duty and in those reporting fewer sleeping hours during the shift (42).

Chronodisruption was described with acute and chronic cardiovascular events. Intramyocellular circadian clock and diurnal variations in fatty acid responsiveness were noted in the rat cardiomyocytes. Reversal of the 12-h/12-h light/dark (L/D) cycle led to re-entrainment of the circadian clock in the heart. Disruption of the circadian clock within the heart via the expression of a dominant negative CLOCK mutant lowered the promotion of myocardial fatty acid-responsive genes during fasting (43). Under pathologic conditions, the endogenous circadian phase corresponds to 10 a.m., the peak time for adverse cardiac events. Diurnal rhythm disruption after myocardial infarction (MI) hinders cardiac healing and exacerbates pathological cardiac remodeling. In a mouse model of MI, a short-term diurnal disruption adversely affected metabolism while altering innate immune responses shown by differences in cytokines, cardiac myeloperoxidase, and macrophage and neutrophil infiltration. Clock mutant mice showed changed infiltration after MI, which is linked to the innate immune responses required for scar formation and associated with reduced blood vessel formation in the infarct region, increased left ventricular dilation, and infarct expansion (44).

In an animal study, the likelihood of ventricular fibrillation (VF) was assessed by the time of the day during which acute coronary failure occurred. A coronary failure between 15.30 and 18.00 led to irreversible VF and death while modeling a similar condition from 11.00 to 15.00 was not associated with VF (45). In a study of 268 consecutive healthy subjects, FMD was diminished in early compared to late morning post-waking hours and predicted long-term cardiovascular events in healthy subjects with no known heart disease (46). FMD of the brachial artery analyzed three times a day (6:30 a.m., 11:30 a.m., and 9 p.m.) in patients with idiopathic dilated cardiomyopathy was lower during the day, manifesting reduction of the normal circadian variation in endothelial function (47).

A meta-analysis encompassing more than 2 million people, described a moderately increased risk for MI and ischemic stroke among shift workers (48). Another chronobiological characteristic of cardiovascular diseases is exhibited in the varying prevalence of adverse cardiovascular events throughout the day. MI, ischemic strokes, and sudden cardiac death (SCD) have higher incidences in the morning hours, corresponding with an increase in the sympathetic tone, peripheral arteries resistance, platelet aggregability, decreased parasympathetic tone, and endothelial function (39).

### Chrono Disruption Is Associated With Heart Failure

Disruption of the circadian rhythm contributes to the pathogenesis of HF (49). In HF, the dynamics are associated with an increased parameter of the scaling exponent of the inter-beat interval. In these patients, the peak in the scaling exponent at the circadian phase corresponds to the time of increased heart vulnerability. The endogenous circadian-mediated effects on cardiac regulation are linked to a day/night pattern of adverse cardiac events (50). The heart rate variability (HRV) and mean arterial pressure (MAP) variability were decreased in rats with HF; this decrease was accompanied by disturbances in the normal circadian pattern of HR and BP (51). Differences were demonstrated in the circadian outcome of angiotensin converting enzyme inhibitors (ACEis) on BP in rats with HF (52). The circadian clock contributes to regulation of the mitochondrial metabolism and maintaining the cardiac function. Ablation of the Bmal1circadian clock gene is associated with mitochondrial defects in the heart including morphological changes and reduced enzymatic activities within the respiratory complex, reduced expression of cardiac genes associated with the tricarboxylic acid cycle, fatty acid oxidative pathway, and mitochondrial respiratory chain. These changes were associated with the development of HF. Similar changes were noted in mice exposed to the chronic reversal of the L/D cycle and disrupted circadian rhythmicity (53). In a study of 1,401 asymptomatic subjects in the Cardiovascular Health Study with interpretable 24-h baseline Holter recordings, irregular HRV parameters were related with CHF. Combined with higher incidence of PVCs, HRV enhanced the predictive power of the Health ABC score (54). Autonomic dysfunction quantified HRV parameters characterized subjects with no benefits from cardiac resynchronization treatment, suggesting that pre-implant HRV study helps in improving qualifications for this treatment. In a study of 719 subjects with normal sinus rhythm enrolled in MADIT-CRT (Multicenter Automatic Defibrillator Implantation Trial-Cardiac Resynchronization Therapy), followed for over 3 years, 124 patients reached the primary end point of heart failure or death and 47 died. In multivariate analysis, low SDNN or low VLF was related to a significantly increased risk of HF and mortality (55).

A blunt BP circadian rhythm in HF was documented in humans and linked to the disappearance of circadian variation in atrial natriuretic peptide (56) and in normotensive patients, with the degree of left ventricle function impairment (57). A narrower decrease in the nocturnal systolic BP correlated with a lower ejection fraction. Respiratory function also varies between the light and dark periods, and these L/D variations were shown in HF. In an animal model, HF was associated with blunted L/D differences in resting and chemoreflex breathing, suggesting that the HF disrupts cardiovascular and respiratory circadian rhythms. Cyclic variation of heart rate (CVHR) is linked with sleep-disordered breathing reflecting cardiac autonomic responses to apneic/hypoxic stress. Blunted CVHR in a night-time Holter ECG predicts increased mortality risk in patients with post-MI, end-stage renal disease, and HF. In a study of 717 patients after MI, decreased cyclic variation amplitude was a predictor of mortality (58). Diurnal variations in respiration and arterial BP were abolished in HF with reduced ejection fraction (HFrEF) (59). Circadian oscillations in calcineurin-dependent activities in the left ventricle of normal mice showed that calcineurin-dependent transcript levels and nuclear occupancy of the nuclear factor of activated T-cells fluctuate over the course of a day, peaking in the morning when mice enter a period of rest (60).

SCD, a prominent cause of death in patients with HF, exhibits diurnal variation and is linked to irregularities in the duration (short or long QT syndromes and HF) or pattern (Brugada's syndrome) of myocardial repolarization. Cardiac ion-channel expression and QT-interval duration, which reflects myocardial repolarization, manifest endogenous circadian rhythmicity controlled by a clock-dependent oscillator, Krüppel-like factor 15 (Klf15). Klf15 transcriptionally regulates a rhythmic expression of the Kv channel-interacting protein 2, which is necessary for the transient outward potassium current (61).

Overall, these data suggest a possible association between HF and alteration in the normal circadian rhythms, implying that novel treatment strategies for HF must take into consideration the timing of treatment administration, adjusting it to the individualized circadian rhythm of patients.

## The Autonomic Nervous System Regulates the Circadian Disruption in HF

The ANS underlie some mechanisms associated with CHF. The sympathetic nerve fibers travel alongside the coronary arteries and terminate in the sub-epicardium. Its activation results in accelerated HR, augmented contractility, and increased afterload. In contrast, the parasympathetic system, which branches from the vagal nerve to the sub-endocardium, slows the HR (62). Chronic sympathetic overactivation was described in HF and diuretic resistance and is related to components of the metabolic syndrome, such as obesity, dyslipidemia, BP elevation, and reduced fasting glucose with hyperinsulinemia (63, 64).

**Figure 1:**
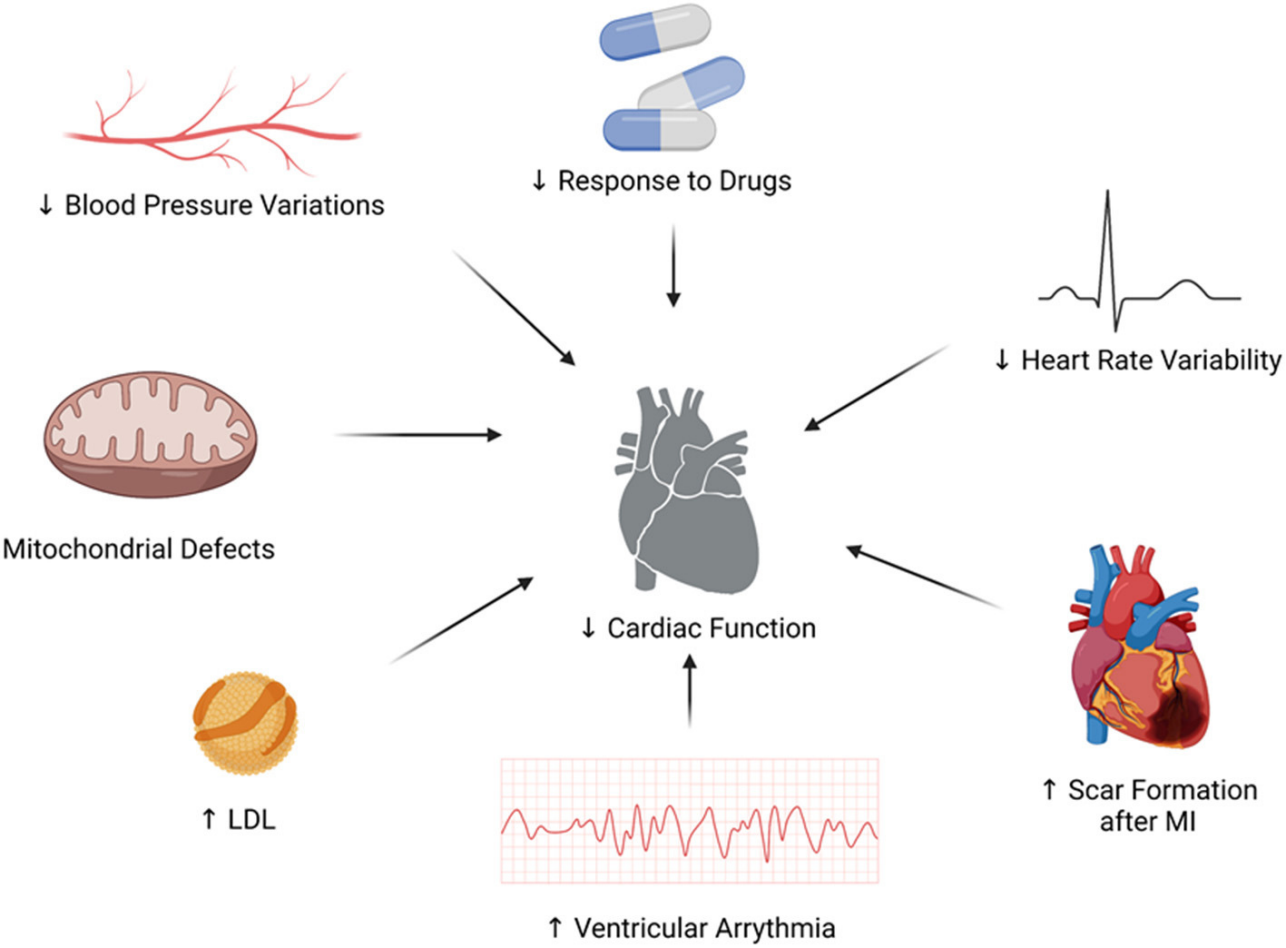
The interfaces between the circadian rhythm and the autonomic nervous system, contributing to the pathogenesis of congestive heart failure.

Figure 1 describes the interfaces between the circadian rhythm and the autonomic nervous system, contributing to the pathogenesis of congestive heart failure.

### The Role of Sodium in Circadian Rhythm

Dietary sodium affects the circadian oscillators downstream of the master light-dark-adjusted pacemaker in the SCN. The chronobiology of renin activity (RA), BP, and fractional excretion of renal sodium (UFENa) and potassium (UFEK) handling in relation to meal timing was studied in dogs. Data showed that RA, UFENa, UFEK, diastolic and systolic BP fluctuate with a circadian periodicity in dogs fed at 07:00 h with a regular diet. Modeling using a fixed 24-h period reflected the variations of UFEK, RA, and BP, and cyclic changes in UFENa suggested a postprandial sodium excretion and a monotonous decay. A delay in the feeding schedule by 6- or 12-h was associated with a shift of comparable magnitude in the rhythm of these biomarkers (65).

### Diurnal Rhythm of the ANS Affect Cardiac Parameters

The diurnal rhythm in the standard deviation of the averages of normal R-R intervals (SDANN) and LF/HIF ratio is disrupted in MI. L/D differences in the LF/HIF ratio change from negative to positive values along with a decrease in SDANN, HR, LF/HIF ratio in the dark phase, and elevated plasma norepinephrine levels (66). These data suggest that the timing of the disturbance of diurnal rhythm in SDANN and the LF/HIF ratio are different from those in HR and in the plasma norepinephrine levels (67). In patients with HF, arterial underfilling caused by decreased cardiac output or peripheral arterial vasodilation activates the SNS, RAAS, and non-osmotic vasopressin release (68). Excess activation of the SNS, which aims to maintain cardiac output, has deleterious effects on the heart in the long run. Activation of the SNS in HF results from interactions between the appropriate compensatory reflexes to pathologic excitatory stimuli associated with the depressed systolic function and additional comorbidities. These interactions elicit adrenergic activation in excess to homeostatic requirements (62). To counteract the increased SNS activation, vagal nerve stimulation was suggested as a novel strategy. However, although profound anti-arrhythmic effects were exerted and cardiac function in HF models was improved (69), this method failed to reduce mortality and disease progression in HFrEF patients in the INOVATE-HF trail (70).

### The Circadian Rhythm of ANS Affects Heart Rate and Blood Pressure

The circadian pattern of HR and BP, which are affected by the ANS, is disrupted in patients with HF. In an animal model of MI, the association between vagal nerve activity and stellate ganglion nerve activity was documented. A circadian variation following MI reached a peak at a time when sympathetic activation was the uppermost and vagal activity was the lowest (71). The circadian and short-term regulation of BP and HR were shown to be preserved in young, non-failing beta1-transgenic mice, suggesting that the loss of blood pressure and HRV in HF cannot be attributed to over activity of the sympathetic system. However, it reveals loss of adrenergic responsiveness to changes in the activity of the ANS (72). Angiotensin II participates in abnormal autonomic cardiovascular control, which occurs during HF progression. In a model of post-MI, HR increased with the severity of HF, loss of circadian HR, MAP, and BRS rhythms were noted, along with an upregulation in the central angiotensin II type 1 receptors (AT1R) and gp91 proteins in the brainstem. Losartan reduced AT1R expression in daytime but failed to restore circadian variability (73). HF is associated with increased LF constituents of diastolic BP variability, an index of sympathetic tone, during the awakening period compared to during the sleeping period. Amiodarone suppressed this transient increase in LF power during the awakening period (74). Circadian changes in ANS function were also observed at the molecular level. Rats with coronary artery ligation-induced HF showed a shift in the adrenoreceptor beta 1:beta 2 ratio toward beta 2 and decreased beta 1 adrenergic stimulation by adenylyl cyclase. These findings were in association with disturbed circadian patterns in BP and HR (75).

### Heart Rate Variability as a Measure of the Association Between ANS and Heart Failure

HRV is a method to visualize the link between ANS and circadian dysfunction to the development of HF. It measures irregularity of intervals between adjacent heartbeats, representing a neurocardiac function and ANS regulation state (76). Normal HRV presents a circadian pattern with parasympathetic parameters peaking at night-time and shows sleep-stage dependence (77, 78). HRV alteration is associated with numerous disease states and correlates with cardiovascular and all-cause mortality, mainly in patients with established cardiovascular disease (79). A decrease in the HRV was associated with increased mortality and was a better predictor of death than conventional clinical management (80). HRV parameters are altered in both animal models of HF and in humans. In a study determining autonomic parameters 3 and 7 weeks after left coronary artery ligation in rats, LF and HF parameters of HRV were reduced in CHF 3 weeks after infarction, in addition to the prolonged loss of baroreflex sensitivity (BRS). Correlation between HRV and MAP variability in the LF domain was reduced in HF (81). HRV and BRS are severely affected in patients with ADHF and improve with clinical stabilization (77–80, 82). In addition, HRV parameters are abnormal in stable patients with HF. HRV parameters were linked to the incidence of CHF in 1,401 asymptomatic, older adults (54). Increased sympathetic activity in CHF is associated with obstructive and central sleep apnea (SA). In a study of patients with CHF and SA, a lowered cardiac autonomic modulation across the 24-h period was documented. The RR variance, LF, HIF parameters of HRV, and spontaneous BRS were reduced in subjects with SA. The HIF power, a marker of vagal activity, elevated during sleep in patients without SA, whereas it did not alter across the 24-h period in subjects with SA. In a study of 167 patients with CHF with central sleep apnea (CSA) and obstructive sleep apnea (OSA), morning premature ventricular contractions and non-sustained VT were more frequent in CSA. CSA was linked to the occurrence of VT irrespective of sleep/wake status (83–85).

Patients with CHF manifested with lower RR interval complexity and loss of its circadian rhythm, along with reduced frequency-domain RR interval variability and its irregular circadian rhythm (86). The circadian variability of RR and QT intervals is altered in CHF due to neurohumoral activation and functional and structural remodeling of the heart. In a study of 121 patients with HF in the sinus rhythm, all subjects showed marked circadian rhythms in the QT, RR, and QTc intervals and the QT/RR slope by cosine-curve fitting. The increased HR was associated with longer QT interval, and steeper QT/RR slope, lowered circadian variability of QT interval and later maximum RR interval were related to increased cardiac mortality (87).

As sympathetic activation underlies the pathogenesis of HF and diuretic resistance, the selective reduction of the renal afferent and sympathetic efferent nerves was proposed to improve diuretic resistance, CHF, and cardio renal disorders (63). Treatments directed against neurohormonal compensatory mechanisms, such as losartan, spironolactone, and beta-blockers, resulted in an improved HRV profile (88, 89). The favorable effect of spironolactone on the ANS showed a circadian pattern and was limited to the morning hours. Patients with decompensated HF on beta-blockers had a higher power in all HRV frequency bands but were significantly associated with low circulating norepinephrine levels (90). Altering effect of these drugs on the circadian pattern of HRV is known in ischemic heart disease (91).

Implantation of a left ventricular assist device (LVAD) can increase the reduced HRV, which correlates with the degree of ventricular dysfunction. HRV was lower in LVAD patients early after device implantation, suggesting that cardiac dysautonomia is still evident in the first 2 months (92). The algorithm provided by LVADs shows circadian variation. In a study of 18 patients with fixed rotation speed continuous-flow LVAD support, the motor current was lower during the night and higher during the day (93).

Despite these supportive data, other studies failed to support circadian variation in patients with HF. Analysis of data from the PRAISE trial showed that SCD in HF did not show an a.m. peak, signifying that circadian sympathetic activation did not affect these events (94). A study of eight patients with mild-to-moderate HF showed that circadian variation of the cardiac autonomic nervous activity (CANA) was preserved. The CANA was affected by patient position and the time of day. Circadian variation was noted in the recumbent position. The HIF power was lower and LF/HIF was higher in the morning than at night (95).

Overall, the currently available data support the role of the ANS in regulating the heart function under circadian rhythm in health and disease. As many of the effective treatments in HF aim to antagonize the neurohormonal compensatory mechanisms, a personalized approach based on understanding the ANS characteristics and circadian rhythm changes may confer clinical benefits.

## Chronotherapy-Based Therapy for CHF

Chronobiology impacts the effectiveness and toxicity of drugs and is linked to the pharmacodynamics and pharmacokinetics of medications. Chronotherapy is based on linking the absorption, metabolism, and elimination of medications to the circadian patterns (96) and involves the use of a specific drug at the most optimal time, pattern, and dose possible, with the goal of maximizing efficacy and minimizing toxicity. The ideal timing is based on circadian-rhythm-dependent biological systems, which alter drugs bioavailability, pharmacokinetics, and pharmacodynamics (97, 98). Identification of a rhythmic marker for selecting the dosing time has been suggested to improve the response to drugs (99).

Table 1 summarizes examples of the linkage between administration time and effect in cardiovascular drugs.

**Table 1:** Examples of the linkage between administration time and effect in cardiovascular drugs.

**Drug** ^**a**^	**Timing**	**Effect**	**References**
ACEi	Evening vs. morning	Reduction in treatment-related cough	(100)
Antihypertensives	Bedtime vs. awakening	Improved blood pressure control and decrease in major cardiovascular events	(101)
Aspirin	Bedtime vs. awakening	Decrease in early morning platelet activity	(102)
Captopril (rats)	Sleep vs. wake time	Effect on cardiovascular remodeling was achieved only when administered during sleep	(103)
Furosemide (rats)	10 a.m. vs. 10 p.m.	Increased urinary volume and sodium exertion	(104)
Torsemide	Bedtime vs. awakening	Improved blood pressure control and 24-hour therapeutic duration	(105)
Valsartan	Bedtime vs. awakening	Greater reduction in proteinuria, delayed decline in GFR and reduced risk of MI	(106)

a *Administered to human subjects unless otherwise stated. ACEi, angiotensin converting enzyme inhibitors; GFR, glomerular filtration rate; MI, myocardial infarction*.

Chronotherapeutic benefits of antihypertensive medications have been demonstrated (107). A study of 19,084 hypertensive patients treated with BP-lowering drugs at bedtime, as opposed to upon wakening, reported improved BP control and cardiovascular outcomes, including MI, HF, and cardiovascular death (101). Low-dose aspirin administered at bedtime compared with during the awakening hours can improve platelet inhibition during the critical morning hours (102). Several studies have supported the chronotherapeutic attributes of loop diuretics. Administration of furosemide to rats at 10 a.m. showed greater urine volume and urinary excretion of sodium and furosemide than that at 10 p.m. A correlation between the urinary output of furosemide and urinary volume of sodium, representing the known mechanism of furosemide, has been described (104).

Pretreatment with clorgyline, a monoamine-oxidase inhibitor that alters circadian variations in the SNS, diminished the variability in furosemide effect (108). Pretreatment with propranolol also caused a loss of variability, while atenolol did not cause the same effect, suggesting the involvement of the beta2-adrenergic receptor (109). In two additional studies, continuous infusion of norepinephrine and denervation of the kidneys caused the same influence of diminished variability (110, 111). In humans, the efficacy of furosemide was improved by bedtime dosing as compared to dosing upon awakening. The fraction of patients with controlled ambulatory BP following therapy was also increased after bedtime treatment (105).

Chronotherapy can control the RAAS overactivation in HF. The efficacy of ACEi in HF depends on the time of administration. ACEis were more effective in preventing heart remodeling when administered during sleep (103). Dosing of ACEi in the evening, at an inactive period, was associated with an improved protective effect against heart hypertrophy in hypertensive rats and also proposed to reduce the severity of the drug-related dry cough of hypertensive subjects treated in the morning (100). Bedtime administration of valsartan normalizes the circadian rhythm and protects the kidneys and heart in patients with CKD (106). An integrated pharmacokinetic-pharmacodynamics model was designed based on the kinetics of the drug and time-varying changes of RAAS, showing that the optimal efficacy of ACEis is achieved with bedtime dosing (112).

Melatonin determines the circadian physiology, and its circulating levels vary on a daily cycle, allowing the regulation of circadian rhythms of multiple biological systems. It was shown to exert cardioprotective effects impacting the clinical course of HF. In a study of 32 patients with CHF, nocturnal melatonin secretion negatively associated with N-terminal pro-brain natriuretic peptide (NT-proBNP). Higher melatonin levels were noted at 02:00 than at 07:00 (113).

Rev-erb alpha, which is the nuclear receptor 1D1, is a circadian rhythm regulator that controls inflammation and glucose and lipid metabolism. Administration of SR9009, a Rev-erb, in a mouse model of MI and HF improved the survival rates and reduced the left ventricular function. These effects were associated with reduced BNP levels and reduced expression of inflammatory-related molecules, such as matrix metallopeptidase 9, Ly6g, Cd11b, IL-6, Mcp1, and phosphorylated NF-kappaB p65, phosphorylated extracellular signal-regulated ligand, and phosphorylated p38. This treatment also reduced neutrophil and pro-inflammatory macrophage infiltration (114). SR9009 improved the long-term cardiac repair post-myocardial ischemia-reperfusion injury in animals. A single therapy decreased cardiac NLRP3 inflammasome, fibroblast, and immunocyte infiltration, in turn augmenting the MI healing process (115).

Taken together, these studies suggest that chronotherapy, including the targeting of ANS activation, exerts a beneficial effect on HF and diuretic resistance-related parameters.

## Inter- and Intra-Patient Variability Affect the Response to Medications

Variability is inherent to biological systems and has been proposed to be part of the normal function of cells and organs (116–119). Variability at the genome and cellular level (118, 120), HRV (121), respiratory variability (122, 123), and gate variability (124) are some examples. The loss or alteration in the physiologic variability is associated with disease states and poor prognoses (125–127). At the cellular level, the cell is packed with a non-uniform spreading of macromolecules that interact specifically and nonspecifically with a drug. This leads to a high degree of variability between individual cells in the response to drugs (128). Many of these variabilities do not follow specific patterns or rules and are characterized by marked inherent intra- and inter-patients unpredictability.

Variability in the heart function was the basis for the development of the heart-slowing medication ivabradine. The sinus node is the central cardiac pacemaker, and its function is associated with several ionic transporter circuits that can produce a rhythm. If one apparatus fails, another one can take over the task. Eliminating a transporter that could carry as much as 80% of the ionic current required for producing the rhythm alters the incidence by only around 10–15%, and this is due to a substitution mechanism. This supports the concept of a high degree of molecular-level variations as a protective mechanism (129, 130).

High degrees of both inter- and intra-patient variability are described for the response to many drugs. There are marked intra- and inter-patient variabilities in drug pharmacodynamics associated with the loss of an effective response to drugs (119, 131–136). For example, high tacrolimus intra-patient variability was associated with graft rejection (134), and intra-patient variability in many antiepileptic circulatory levels in stable patients was described with observed differences of tens of percentages in the serum levels (137).

Regular dosing regimens for diuretics are not compatible with physiological variability and may further increase drug resistance. Taking a constant dose of diuretics on a regular basis, or continuously increasing the dose, may increase the serum drug levels reaching a peak level and its subsequent gradual decrease. This process is observed to repeat on a daily basis. It is a monotonic cyclic pattern associated with adaptation to the effect and results in the partial or complete loss of the response to the diuretic (12–14). Part of the loss of the effect is associated with augmentation of the compensatory responses to diuretics as a result of a continous increase in doses, which further reduces their effect. For several drugs, a regular dosing regimen or continuous increase of a dose was proposed to contribute to drug resistance, compared with the irregular consumption of the same dose or with altering the daily dose (117, 138).

A model for heartbeat was developed built on a noise titration assay, which provides a time-resolved and quantitative degree of the chaos. It uses the HIF and LF parameters of HRV for determining whether they reflect stochastic or chaotic phenomenon (139). Noise titration of the running short-segment Holter tachograms from healthy people showed circadian sleep/wake-dependent heartbeat chaos that linked with the HF parameter (respiratory sinus arrhythmia). In contrast, in patients with HF, a near-regular heartbeat was non-chaotic and resulted in transient chaotic rhythms. HRV alterations in HF were accompanied by little changes in approximate entropy, a measure of signal irregularity, which reflects an autonomic, cardiac, respiratory, and circadian/sleep-wake changes (139).

A model using time irreversibility analysis was developed for the analysis of the short heart period sequences derived from 24 h Holter recordings. Irreversible dynamics over short time scales were noted, while the irreversibility of longer time scales was marginal. In healthy subjects, the percentage of irreversible dynamics was higher during the daytime than during the night-time, suggesting augmented non-linear dynamics during the daytime. In healthy subjects, the non-linear behavior reflected that bradycardic ones run shorter than the tachycardic ones during the daytime. In contrast, patients with HF demonstrated an increased percentage of irreversible series along with a reverse pattern, showing the tachycardic ones run shorter than the bradycardic ones (140).

## Establishing a Chronotherapy and Variability-Based Algorithm for Improving the Response to Effectivenss of Diuretics

Using treatment regimens based on aperiodic intervals and at irregular strengths has been suggested to improve response to diuretics. For maximum benefits, irregular, pulsed, or multiple intervals-based administration of a chronic drug at continually changing dosage strengths may improve the overall effect, thereby reducing the likelihood of drug resistance (117, 119, 141). Manipulating the conditions of living organs using rhythmic administration of altered feeding schedules or several drugs appears successful (99, 142).

Establishing a novel model for overcoming diuretic resistance was proposed based on a closed loop system, which comprises clinical, laboratory, and sensors-derived inputs, including PA pressure sensors. Alteration of rhythmicity may adversely affect homeostatic regulation and lead to deleterious effects. These changes in therapeutic regimens should take into account the inter- and intra-patient variability, alterations of the biological clock, and ANS responses.

This model has been developed at four levels. In the first step, random alterations in the dosing and timing of administration are introduced into the regimens. The implementation of irregular regimens is expected to improve the response to diuretics by lowering the harmful effects of compensatory mechanisms (117, 138, 143, 144). The physician is asked to register the patient and to enter into the systme the ranges for the dosages and for the times of administration. Patients receive daily reminders for taking the drug. The algorithm randomly alters the dosages and times of administration on a daily basis (143, 144).

In the second step, an increase in the efficiency of diuretics is achieved by administering drugs that are at times the best tolerated by synchronizing medication concentrations to rhythms in disease activity. Technologies for drug delivery precisely in a time-modulated mode by bedside or ambulatory pumps can improve both the safety and efficacy of the chronic use of diuretics (99).

**Figure 2:**
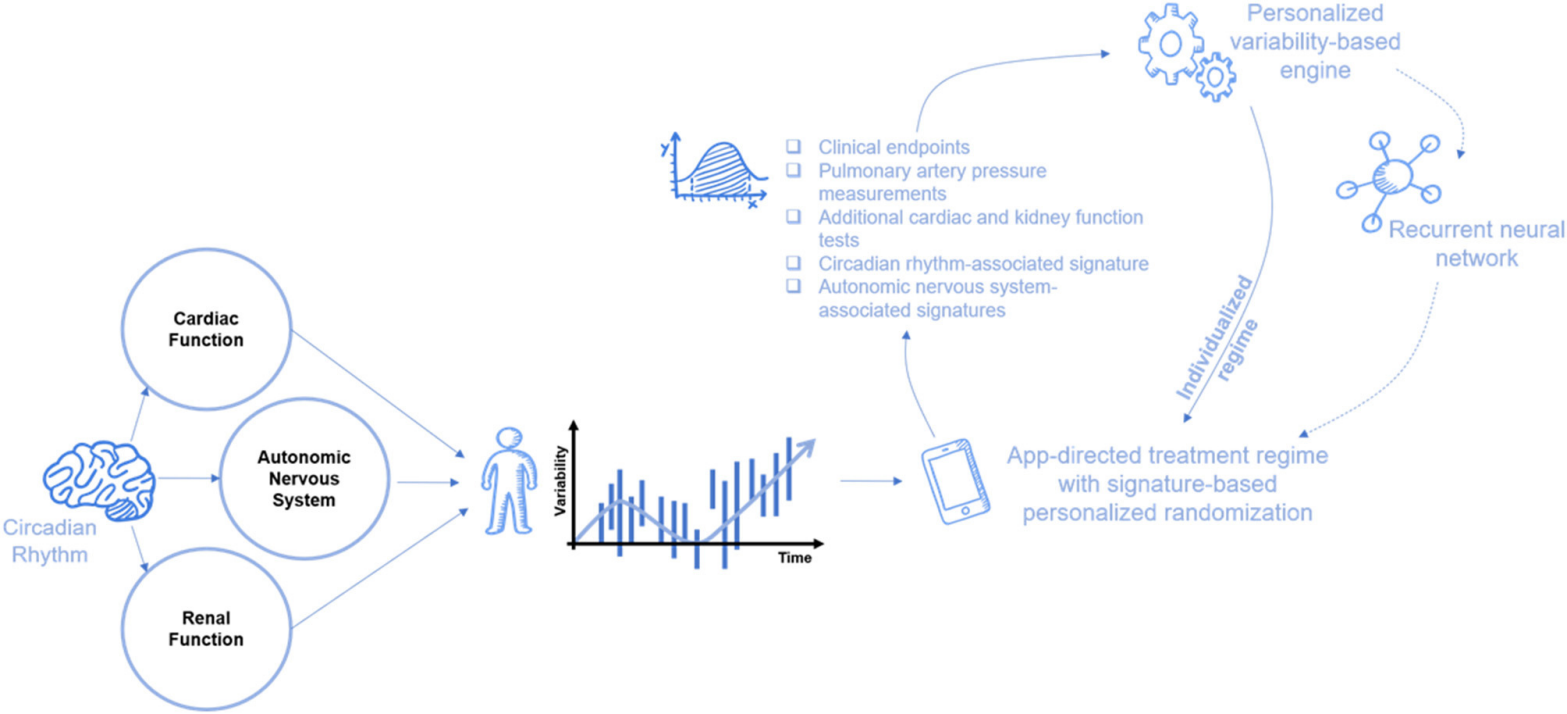
A suggested scheme of the closed loop machine learning system for improving the response to diurectis in patients with HF. Inputs from various sensors are being incorporated in a dynamic manner for determining the timings of administration and dosages in a personalized approach. In a dynamic changing system with multiple alternating parameters, a continuously changing dosing regimen is generated based on the individualized-variability pattern that comprises data from the ANS (e.g., HRV), heart, and kidney. Cardiac, renal, and ANS, along with numerous other physiological and biological processes, exhibit intrinsic randomness. These variations in their activity are also contributed from the generalized circadian rhythm and from intrinsic biological rhythms. Randomized treatment administration within the preset limits augmented with personalized signature from the clinical status, echocardiographic, HRV, pulmonary artery pressure monitoring, biomarkers, and more can improve outcomes of the diuretic treatment. In addition, randomization might prevent renal adaptivity involved in the development of diuretic resistance. ANS, autonomic nervous system; HRV, heart rate variability.

In the third stage, machine learning is introduced to regulate the variability and chronotherapy-based regimens using closed loop systems that control the dosing and timing in an individualized manner. This enables the overcoming of intra- and inter-patient variability, which prevents the “single bullet for all” concept from being applicable to the majority of patients. This concept is being introduced for overcoming drug resistance in several chronic diseases (142, 143, 145–156). In the last step, signatures based on parameters relevant to the pathogenesis of HF, mechanisms of action, and the cardio renal axis are introduced in a personalized approach for tailoring the appropriate dosing in a continuous and consistent manner (143, 144) (Figure 2).

In summary, CHF remains a major clinical problem with an enormous morbidity and mortality burden. Diuretic resistance is a major obastacle for the effective long-term treatment of HF with considerable inter- and intra-patient variability, in addition to the implication of both the ANS and chronobiology in the pathogenesis and progression of HF. A personalized machine learing algorithm, which comprises continously changing parameters derived from clinical, laboratory, and sensors-derived inputs, including inputs from PA measurements, is suggested as an effective tool for improving the resposne to duretics. Ongoing trials will determine if these platforms are efficacious in reducing the adverse clinical outcomes and improving long-term prognoses.

## Author Contributions

All authors listed have made a substantial, direct and intellectual contribution to the work, and approved it for publication.

## Conflict of Interest

YI was the founder of Oberon Sciences. The remaining authors declare that the research was conducted in the absence of any commercial or financial relationships that could be construed as a potential conflict of interest.

## Publisher's Note

All claims expressed in this article are solely those of the authors and do not necessarily represent those of their affiliated organizations, or those of the publisher, the editors and the reviewers. Any product that may be evaluated in this article, or claim that may be made by its manufacturer, is not guaranteed or endorsed by the publisher.

## Abbreviations

CHF: congestive heart failure
HF: heart failure
ANS: autonomic nervous system
NKCC: sodium-potassium-chloride symporter
RASS: renin-angiotensin-aldosterone system
CKD: chronic kidney disease
OAT: organic anion transporter
SNS: sympathetic nervous system
ADHF: acute decompensated heart failure
PA: pulmonary artery
QALY: quality-adjusted life years
SCN: suprachiasmatic nucleus
CLOCK: circadian locomotor output cycle kaput
BMAL1: brain and muscle ARNT-like 1
Per: period
Cry: cryptochrome
BP: blood pressure
HR: heart rate
L/D: light/dark
HFrEF: heart failure with reduced ejection fraction
MI: myocardial infarction
SCD: sudden cardiac death
HRV: heart rate variability
MAP: mean arterial pressure
ACEi: angiotensin converting enzyme inhibitors
Klf15: kruppel-like factor 15
SNS: sympathetic nervous system
LF: low frequency
HIF: high frequency
BRS: baroreflex sensitivity
OSA: obstructive sleep apnea
CVHR: cyclic variation of heart rate
FMD: flow-mediated dilation
SA: sleep apnea
LVAD: left ventricular assist device
SDANN: standard deviation of averages of normal R-R intervals
AT1R: angiotensin II type 1 receptor
RA: renin activity
Una, fe: fractional excretion of sodium in the urine
UK, fe: fractional excretion of potassium in the urine
CANA: cardiac autonomous nervous activity
CSA: central sleep apnea.
